# Unveiling Conserved Molecular Pathways of Intramuscular Fat Deposition and Shared Metabolic Processes in Semitendinosus Muscle of Hereford, Holstein, and Limousine Cattle via RNA-Seq Analysis

**DOI:** 10.3390/genes16080984

**Published:** 2025-08-21

**Authors:** Saideh Eskandri Nasab, Gholam Reza Dashab, Mohammad Rokouei, Zahra Roudbari, Tomasz Sadkowski

**Affiliations:** 1 Department of Animal Science, Faculty of Agriculture, University of Zabol, Zabol 98615-538, Iran; saideh.skandary68@gmail.com (S.E.N.); dashab@uoz.ac.ir (G.R.D.); roukouei@uoz.ac.ir (M.R.); 2 Department of Animal Science, Faculty of Agriculture, University of Jiroft, Jiroft 78671-55311, Iran; 3 Department of Physiological Sciences, Institute of Veterinary Medicine, Warsaw University of Life Sciences, 02-787 Warszawa, Poland

**Keywords:** adipogenesis, cattle breeding, hub genes, intramuscular fat, marbling, transcriptome

## Abstract

Background: Intramuscular fat (IMF) enhances marbling, improving meat quality and value. Transcriptome analysis enables the identification of genes and pathways involved in IMF deposition, supporting targeted breeding and nutritional strategies to improve beef quality. Methods: This study used RNA-Seq to compare gene expression in high- (Hereford; Her), moderate- (Holstein Friesian; Hf), and low-marbling (Limousine; Lim) Semitendinosus muscle. Using Illumina’s NovaSeqX Plus, sequencing data underwent quality control with FastQC to remove low-quality reads and adapters, followed by alignment to the bovine genome using HISAT2. Differential expression analysis was performed using DESeq2, and genes were filtered based on a threshold of *p*-value < 0.05 and |log2FC| > 0.5 to identify significantly regulated genes. Results: A total of 21,881 expressed genes were detected, with 3025 and 7407 significantly differentially expressed in Her and Hf vs. Lim, respectively (|log2FC| > 0.5, *p* < 0.05). Protein–protein interaction analysis revealed 20 hub genes, including *SMAD3*, *SCD*, *PLIN2*, *SHH*, *SQLE*, *RXRA*, *NPPA*, *NR1H4*, *PRKCA*, and *IL10*. Gene ontology and KEGG pathway analyses linked these genes to lipid metabolism and IMF-associated pathways, such as PPAR signaling, fatty acid metabolism, and PI3K–Akt signaling. Conclusions: These findings highlight RNA-Seq’s utility in uncovering the genetic basis of marbling and the importance of aligning beef production with consumer demands through genetic improvements. This study aimed to identify breed-independent molecular mechanisms of intramuscular fat deposition and shared metabolic processes in the Semitendinosus muscle to improve beef quality.

## 1. Introduction

Historically and from an evolutionary viewpoint, human societies valued meat consumption as a nutritious and profoundly symbolic sustenance, set against a backdrop of biosocial necessities spanning three million years [1]. Globally, meat consumption patterns are shaped by a complex interplay of nutritional needs, health considerations, economic conditions, and environmental concerns. Although global meat production continues to rise, many developed countries have reached a plateau and are now witnessing a gradual decline in consumption. Nevertheless, beef remains a culturally significant and nutritionally valuable part of the diet in many regions, consistently ranking as the third most consumed meat after pork and poultry over recent decades [2]. Although numerous factors can impact the beef grading system, the current approach uses the marbling level as a visual cue for assessing palatability within the beef quality grading framework. Furthermore, compared to visceral and subcutaneous fat depots, the increased quantities of polyunsaturated fatty acids (PUFAs) present in IMF positively impact human health. Hence, producers endeavor to promote cattle trade with sufficient levels of marbling while ensuring that carcass-cutting yields are not compromised [3]. Marbled beef has a significant quantity of protein, and its rich amino acid composition closely matches the human body’s essential requirements. It has been suggested that marbled beef may enhance the body’s ability to grow and develop, repair tissues, and promote overall health [4].

The accumulation of marbling, the IMF that appears as white flecks or streaks within the muscle, in cattle primarily occurs via adipocyte cell hyperplasia and hypertrophy throughout their lifespan. The hyperplasia of IMF cells during the fetal and neonatal stages plays a crucial role in enabling the hypertrophy of IMF cells in later periods by establishing the necessary locations [5]. IMF, deposited between myofibers as marbling, enhances meat’s tenderness, flavor release, and visual appeal. Its distribution reflects a complex quantitative trait influenced by environmental factors (e.g., diet, management) and genetic determinants regulating adipogenesis and lipid metabolism [6]. The impact of cattle breeds or genetic variation on the amount of beef and the fatty acid composition of IMF has been previously documented [7]. Hereford (Her), Holstein Friesian (Hf), and Limousine (Lim) are three widely recognized cattle breeds with distinct characteristics and breeding histories. Holstein cattle, primarily known for their dairy production, have also been studied for beef traits. Hereford and Limousine, on the other hand, are traditional beef breeds with a strong focus on meat quality. Comparative studies of these breeds can shed light on the genetic differences influencing marbling and help identify breed-specific genetic markers for improving beef quality [8].

Transcription factors and secreted molecules regulate gene expression during adipocyte proliferation and differentiation, shaping fat cell number and size. Understanding these molecular mechanisms is key to improving IMF deposition and enhancing meat production in beef cattle [9]. The biological process of marbling development is complex, involving a network of enzymes, hormones, and metabolites that regulate adipocyte deposition and the metabolic pathways of differentiation. The dynamic balance between lipid synthesis and muscle tissue degradation ultimately determines the extent of IMF accumulation. Therefore, examining a broad spectrum of genes, particularly those associated with lipid metabolism and adipocyte differentiation, can offer valuable insights into the molecular drivers of marbled muscle phenotypes and help identify key regulatory elements responsible for IMF development [10]. The analysis of gene expression profiles enables understanding phenotypic distinctions, ramifications, and fundamental evolutionary mechanisms for individual genes [11]. Recent advances have greatly improved RNA-Seq data analysis, making it a robust and reliable tool for quantifying gene expression and identifying novel transcripts. Since gene expression profiles reflect the activity of biological pathways underlying phenotypic traits, transcriptomic analysis enables researchers to link molecular mechanisms to observable characteristics, such as growth, fat deposition, or disease resistance [12,13].

The primary objective of this study was to identify breed-independent, conserved molecular mechanisms of IMF deposition in the Semitendinosus muscle of cattle, based on IMF content variability. This aimed to minimize the role of breed-specific genetic backgrounds and identify genes and molecular pathways associated with IMF deposition, regardless of breed, for improving beef quality in various cattle populations. This comparative approach provides a broader perspective of IMF regulation at the genetic level. It determines targets for marbling enhancement in beef and dairy breeds, with the latter being utilized as a source of beef in certain countries and the European Union, representing 25–30% of beef production [14].

## 2. Materials and Methods

### 2.1. Ethical Statement

This study was conducted under the national and institutional regulations governing the use of animals in research, as stipulated by the Polish Act of 21 January 2005 on the protection of animals used for scientific or educational purposes. In addition, the procedures comply with relevant European legislation, including EU Directive 2010/63/EU on the protection of animals used for scientific purposes. Since tissue sampling was performed during routine commercial slaughter without additional invasive procedures, formal ethical approval was not required.

### 2.2. Animals and Skeletal Muscle Sampling

The animals in each breed originated from three independent herds. After weaning, all animals were raised in a closed herd under uniform feeding conditions at the Institute of Genetics and Animal Breeding, Polish Academy of Sciences, in Jastrzębiec, Poland. From that time until slaughter, they were kept in a loose housing system on this farm and fed ad libitum with a total mixed ration (TMR) composed of 75% corn silage, 20% concentrates, and 5% hay, and had unlimited access to water. At 15 months, all bulls were slaughtered at a local abattoir following a 24 h fasting period. The carcasses were then chilled for 24 h at 4 °C before being dissected [15]. After slaughter, the collected skeletal muscle tissue was immediately cleaned of connective tissue, placed in liquid nitrogen, and stored at −80 °C until analyzed. The experimental design consisted of 15-month-old bulls of three breeds, selected based on their varying IMF content in *Semitendinosus* muscle: high-marbling Her, moderate-marbling Hf, and low-marbling Lim (n = 4 for each breed). The average IMF content was identified by two methods, providing complementary insights into total fat content and visible marbling. The Soxhlet method (PN-ISO 1444:2000 Standard [16]) determined the gravimetric percentage of total extractable IMF (1.10%, 0.81%, and 0.53% for Her, Hf, and Lim, respectively), as previously described [15]. A computer vision system (CVS) analysis provided an area-based estimation of visible IMF (3.23%, 2.61%, and 1.57% for Her, Hf, and Lim, respectively), as previously described [15]. The Soxhlet method served as the quantitative standard for sample selection, while the CVS offered a non-invasive visual characterization of fat distribution. *Semitendinosus* muscle samples with the highest IMF content within breeds were selected for analysis, representing each of the above-described breeds.

### 2.3. RNA Extraction and Sequencing

Total RNA was extracted from skeletal muscle tissue samples using the RNeasy Mini Kit (Qiagen, Germantown, MD, USA), following the manufacturer’s protocol. Briefly, approximately 30 mg of frozen muscle tissue was homogenized in 600 µL of RLT buffer containing β-mercaptoethanol using a rotor–stator homogenizer. The lysate was centrifuged to remove debris, and the supernatant was transferred to a new tube. Ethanol (70%) was added to the lysate to promote RNA binding to the silica membrane of the RNeasy spin column. The sample was loaded onto the column, followed by sequential washing with RW1 and RPE buffers. An on-column DNase I digestion was performed using RNase-free DNase (Qiagen, Germantown, MD, USA) to remove potential genomic DNA contamination. Finally, RNA was eluted in 30–50 µL of RNase-free water. The purity and concentration of extracted RNA were measured using a Qubit fluorometer (Thermo Fisher Scientific, Waltham, MA, USA). RNA integrity was assessed using the TapeStation D1000 (Agilent Technologies, Santa Clara, CA, USA), and a quality control (QC) report was generated. Samples with RIN > 8 (RNA Integrity Number) were included in the further analysis. The VAHTS Universal V10 RNA-Seq Library Prep Kit for Illumina (Vazyme, Nanjing, China) was used for library preparation. Libraries were sequenced on an Illumina NovaSeqX Plus platform with 25B flow cells, generating approximately 100 million paired-end 150 bp reads (PE150) per sample. The resulting raw reads were stored in the FASTQ format, and their quality was assessed based on Q scores using FastQC. The datasets generated during the current study are publicly available in the NCBI Sequence Read Archive (SRA) under accession number: PRJNA1187860.

### 2.4. RNA-Seq Data Processing and Analysis

After performing sequencing, the initial stage of the RNA-Seq workflow is quality control. The quality control procedure must implement measures to guarantee that the raw data meets the specified quality standards. This involves adapter trimming, eliminating sequences not originating from the target organism, and excluding low-quality reads and bases that have not been called. Due to the availability of data generated from the Illumina platform, we employ FastQC (version 0.11.9) to assess the quality of the files. FastQC is conveniently accessible to the public via http://www.bioinformatics.babraham.ac.uk/projects/fastqc/, accessed on 30 April 2024. We subsequently evaluated the alignment based on the bovine genome. Aligning extensive collections of high-throughput sequencing to a specified genome is a fundamental process in examining RNA-seq data. Our investigation utilized the Ensembl release 110 (https://www.ensembl.org/Bos_taurus, accessed on 5 May 2024) genome edition’s Gene Transfer Format (GTF) file as the established reference point. To implement the alignment step, we considered HISAT2 (version 2.2.1), designed to accommodate single- and paired-end reads. It enables alignment speeds up to twice as fast due to its enhanced alignment strategy [17]. The feature counts (version 2.0.1) were used to tally reads that arise from RNA-sequencing. The featureCounts function is designed to process data comprising aligned reads stored in SAM files. Upon execution, featureCounts generates a count matrix organized by genes, which can be readily utilized in gene expression analysis tools, like DESeq2.

### 2.5. Differentially Expressed Genes

Differential gene expression analysis is crucial in processing RNA-Seq data to identify genes exhibiting differential expression patterns across distinct experimental conditions. After normalizing RNA-Seq quantification data, an analysis was conducted to ascertain either upregulated or downregulated genes. Within this study, the differential expression analysis on RNA-Seq data derived from the DESeq2 (version 1.30.1) pipelines was executed using the R programming language. The PCA plot was created by combining functions provided by the R software (version 4.4.1). Various statistical procedures are created for the selection of DEGs. We evaluated and selected the DEGs based on 0.5 < log2FC< −0.5 and *p*-value < 0.05. The genes are uploaded in the DAVID version 2021 (v2023q4, https://davidbioinformatics.nih.gov, accessed on 15 May 2024) bioinformatics resource until the produced genes list those that are most related to marbling. Common genes most related to marbling between Hf/Lim and Her/Lim breeds were identified using a Venn diagram and displayed on the heatmap available at the SRplot website (http://www.bioinformatics.com.cn/srplot, accessed on 20 May 2024).

### 2.6. Protein–Protein Interaction (PPI) Network

To acquire further knowledge regarding the biological activities of the genes in Her/Lim and Hf/Lim, a biological functional enrichment analysis was conducted utilizing the DAVID database. The genes most associated with marbling were identified to evaluate the interactions among genes, and analyses were performed using the web-based application of STRING version 11.5 (https://string-db.org, accessed on 25 May 2024), applying a minimum required interaction score of 0.400. The results were exported as a tsv file and entered into Cytoscape software (version 3.9.1) to visualize the network. Principal component analysis (PCA) was performed before the differential expression analysis to mitigate the potential impact of breed effects and individual variability. Cytoscape is an openly available platform for visualizing molecular interaction networks and integrating data [18]. Following this, the hub gene scores were assessed, and the degree was used to ascertain the central hub gene of marbling. Genes with the highest degree of association were identified as the hub genes.

### 2.7. Functional Analysis (Gene Ontology and Pathway Enrichment Analyses)

GO and pathway enrichment analyses were conducted using the DAVID and KEGG databases (www.kegg.jp/kegg/kegg1.html, accessed on 10 June 2024). The categorization into distinct groups was performed based on their BP, CC, and MF. The enrichment of gene sets about specific GO terms and signaling pathways was ascertained by employing Fisher’s exact test (*p*-value < 0.05), and only those deemed statistically significant were considered. Subsequently, the data were presented using a web-based platform for further scrutiny and illustrated by creating a chart at http://www.bioinformatics.com.cn/srplot, accessed on 10 June 2024.

## 3. Results

### 3.1. Measuring Data Quality

**Figure 1:**
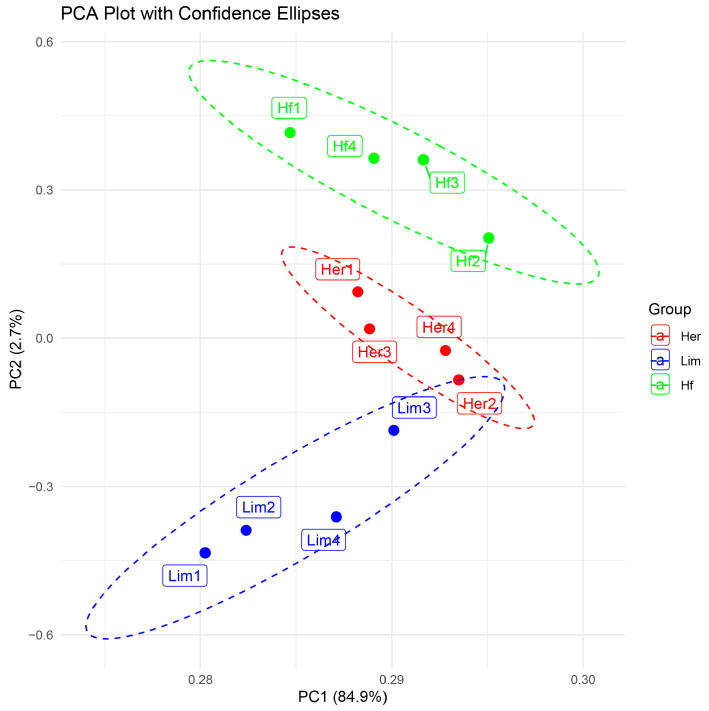
PCA plot showing the clustering of samples based on gene expression profiles. It separates the biological replicates of high-marbling Her and moderate-marbling Hf samples (red and green) from low-marbling Lim samples (blue).

The present study data have been analyzed using FastQC, which assists in the quality control process of the RNA-Seq. All further computational analyses were performed with this data. GC content was within a spectrum of 55 GC%, signifying an exemplary level of data integrity. Examining N-contents’ distribution (number of unread nucleotides) has yielded the most well-documented outcomes. The result zero serves as an indication of good-quality data. In per base analysis, the coverages for the DNA nucleotides and the parallel view between the complementary bases of the low-quality nucleotides were removed. The principal component analysis (PCA) plot enabled quality control analysis by examining the data distribution for each sample. This study showed that samples from the same breeds with similar traits—Herford (Her), Holstein Friesian (Hf), and Limousine (Lim)—clustered closely together. Notably, samples representing different intramuscular fat (IMF) content (Her for high-marbling, Hf for moderate-marbling, and Lim for low-marbling) were distinctly separated. While samples generally clustered by breed, some overlap indicates the presence of shared expression patterns and biological variability across individuals. The proximity of some Herford and Limousine samples is likely due to both being beef breeds, which can share more inherent genetic and physiological similarities compared to dairy breeds. Figure 1 illustrates a correlogram generated between the breeds in the report.

### 3.2. RNA-Sequencing Data Processing

After filtering low-quality reads and aligning them to the bovine reference genome using HISAT2, we obtained mapping rates ranging from approximately 80% to over 97%, depending on the sample (Table 1). On average, more than 87% of clean reads showed unique mapping across different genomic regions. Reads that did not align or aligned to multiple positions were excluded from downstream analyses. The number of properly paired reads and uniquely mapped reads indicates high sequencing quality across most samples. Some variation in the mapping rate exists between samples (e.g., Hf1 showing lower mapping), possibly due to breed-specific genome characteristics, sample quality, or sequencing depth.

The table presents detailed read alignment metrics from the RNA-Seq analysis performed on 12 bovine muscle samples, including four animals from each breed: Limousine (Lim1–Lim4), Hereford (Her1–Her4), and Holstein Friesian (Hf1–Hf4). For each sample, the total number of sequenced reads (total reads) and the number of reads mapped to the bovine reference genome (mapped) are shown. Values are presented in millions (M) of reads and highlight breed-specific alignment efficiency and read pair dynamics. Paired seq indicates the number of reads identified as part of read pairs. Read1 and Read2 refer to the number of forward and reverse reads, respectively. Properly paired denotes read pairs that aligned concordantly. Mate-mapped represents read pairs with mates aligned to the genome. Singletons are reads where only one of the pair was successfully mapped. These metrics confirm high-quality sequencing and successful alignment suitable for downstream transcriptomic analysis.

### 3.3. Analysis of Differential Gene Expression

**Figure 2:**
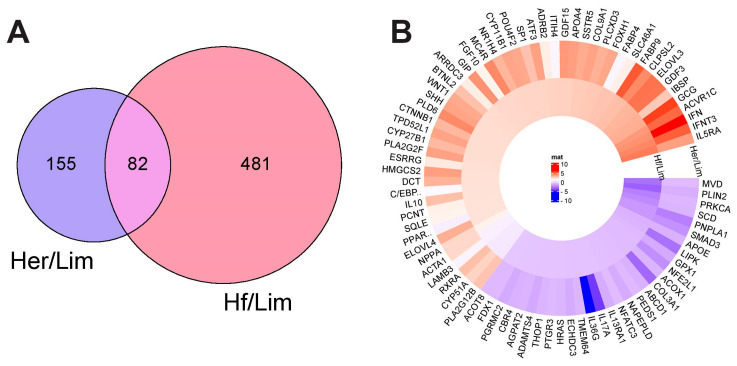
Analysis of gene expression. (**A**) Common genes identified between Hf/Lim and Her/Lim breeds using the Venn diagram. (**B**) The heat map shows expression patterns of 82 common genes in Her/Lim and Hf/Lim comparisons. Each horizontal line represents a gene, with blue indicating similar, lower expression in Her and Hf individuals vs. Lim and red indicating similar, higher expression in Her and Hf vs. Lim (Supplementary Tables S1–S3). Hf—Holstein Friesian; Her—Hereford; Lim—Limousine.

Differential gene expression was analyzed using DESeq2, applying a threshold of *p*-value < 0.05 and |log2FC| > 0.5. Out of 21,881 genes, 3025 were differentially expressed in the Her/Lim comparison (1611 upregulated, 1414 downregulated), and 7407 in the Hf/Lim comparison (6100 upregulated, 1307 downregulated). These findings indicate a stronger transcriptomic response in Hf/Lim. Functional annotation using DAVID revealed 237 marbling-related genes in Her/Lim and 563 in Hf/Lim, with most genes involved in lipid metabolism and adipose tissue development. A Venn diagram (Figure 2A) showed that 82 genes were shared between both comparisons, suggesting conserved molecular mechanisms regulating IMF deposition across breeds. The expression patterns of the 82 shared genes were further visualized using a circular heatmap (Figure 2B), highlighting differences in gene activity across the breeds. Notably, Hf/Lim exhibited higher expression of immune-related genes, such as IFNγ and IL10RA, while lipid metabolism genes, like SCD and PLIN2, were downregulated. These contrasting expression patterns point to breed-specific regulatory mechanisms related to marbling. Hierarchical clustering supported these patterns by grouping samples based on expression intensity. PCA results were also consistent, confirming distinct gene expression profiles between the cattle breeds. Detailed functional results are available in the Supplementary Materials.

### 3.4. PPI Network Analysis

This study identified 30 hub genes with high connectivity in the protein–protein interaction (PPI) network analysis. These include 10 genes from the Her/Lim comparison (high- vs. low-marbling), 10 from the Hf/Lim comparison (moderate- vs. low-marbling), and 10 genes shared between both comparisons.

The hub genes identified in the Her/Lim group were *IL6*, *SMAD3*, *SLC2A4*, *SCD*, *PLIN2*, *SREBF1*, *RXRA*, *NR3C1*, *XBP1*, and *PNPLA2*. For the Hf/Lim comparison, the hub genes included *MYOD1*, *TREM1*, *TBX21*, *SCARB2*, *GPAM*, *VSNL1*, *PNLIP*, *FOXO1*, *TRAF3*, and *DGAT1*. The common hub genes shared between both comparisons were *SMAD3*, *SCD*, *PLIN2*, *SHH*, *SQLE*, *RXRA*, *NPPA*, *NR1H4*, *PRKCA*, and *IL10* (Supplementary Tables S2 and S3).

**Figure 3:**
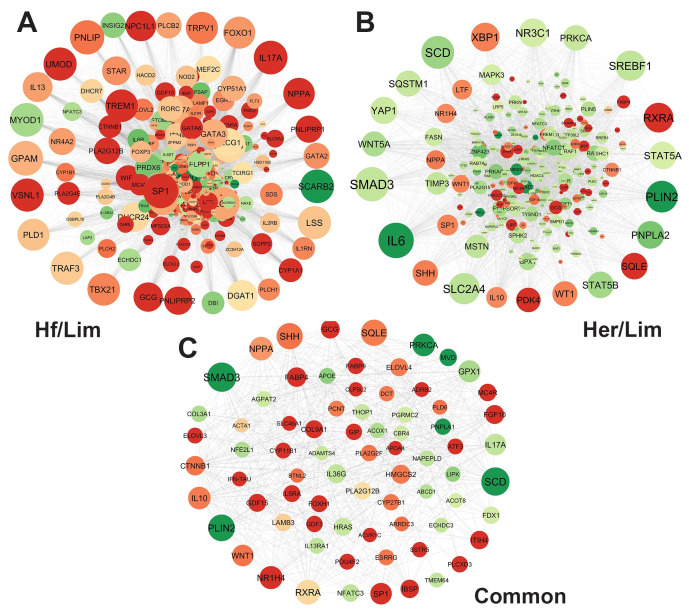
PPI analysis of the most critical genes regulating the gene network in (**A**) Hf/Lim, (**B**) Her/Lim, and (**C**) common breeds using Cytoscape software. The transition in color from green to red indicates increasing log2 fold change (from low to high), and node size is scaled by degree. Hf—Holstein Friesian; Her—Hereford; Lim—Limousine.

In the network visualization (Figure 3), node size represents the degree of connectivity, while color intensity (green to red) reflects the fold change in gene expression. Larger and redder nodes indicate genes with high connectivity and strong differential expression.

These 30 hub genes represent promising candidates for further investigation. Due to their central role in the gene networks related to marbling, they may serve as potential biomarkers. However, functional validation is required to confirm their biological relevance and regulatory significance in intramuscular fat deposition.

### 3.5. Signaling Pathway

**Figure 4:**
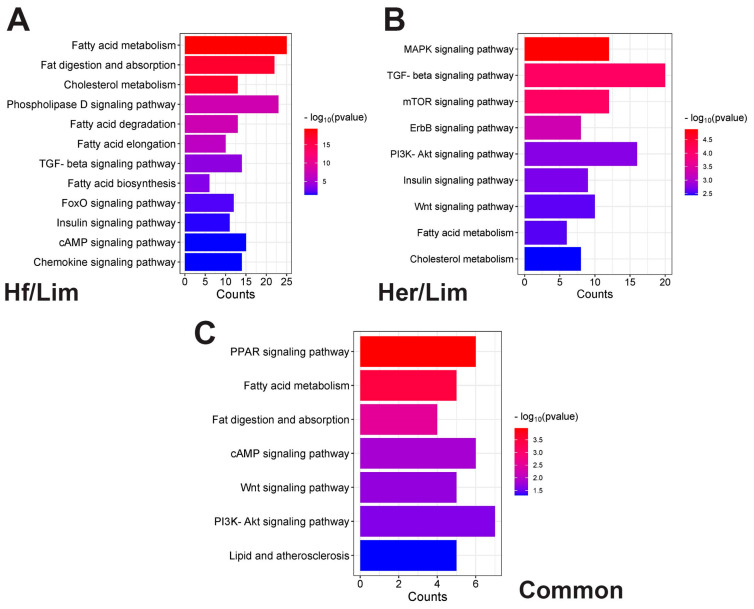
Functional signaling pathway annotations for the DEGs. Bar plots Her/Lim (**A**), Hf/Lim (**B**), and common (**C**) show the top signaling pathway. The lower part shows the number of genes related to the pathways, and the color change in the bars is based on the *p*-value. Hf—Holstein Friesian; Her—Hereford; Lim—Limousine.

The critical signaling pathways regulating IMF were identified (Figure 4; Supplementary Table S4). Bar plots Hf/Lim (A), Her/Lim (B), and common (C) show the top signaling pathway. The lower part shows the number of genes related to the pathways, and the color change in the bars is based on the *p*-value. Using differential gene expression patterns can clarify their roles in beef quality.

In the Her/Lim comparison, these pathways include the MAPK signaling pathway, cholesterol metabolism, fatty acid metabolism, Wnt signaling pathway, ErbB signaling pathway, mTOR signaling pathway, PI3K–Akt signaling pathway, insulin signaling pathway, and TGF-beta signaling pathway.

In the Hf/Lim comparison, fat digestion and absorption, fatty acid metabolism, cholesterol metabolism, Phospholipase D signaling pathway, fatty acid degradation, Chemokine signaling pathway, fatty acid biosynthesis, TGF-beta signaling pathway, fatty acid elongation, FoxO signaling pathway, cAMP signaling pathway, and insulin signaling pathway.

In the common comparison, the PPAR signaling pathway, fatty acid metabolism, fat digestion and absorption, cAMP signaling pathway, Wnt signaling pathway, PI3K–Akt signaling pathway, and lipid and atherosclerosis regulate IMF development.

### 3.6. Gene Ontology

**Figure 5:**
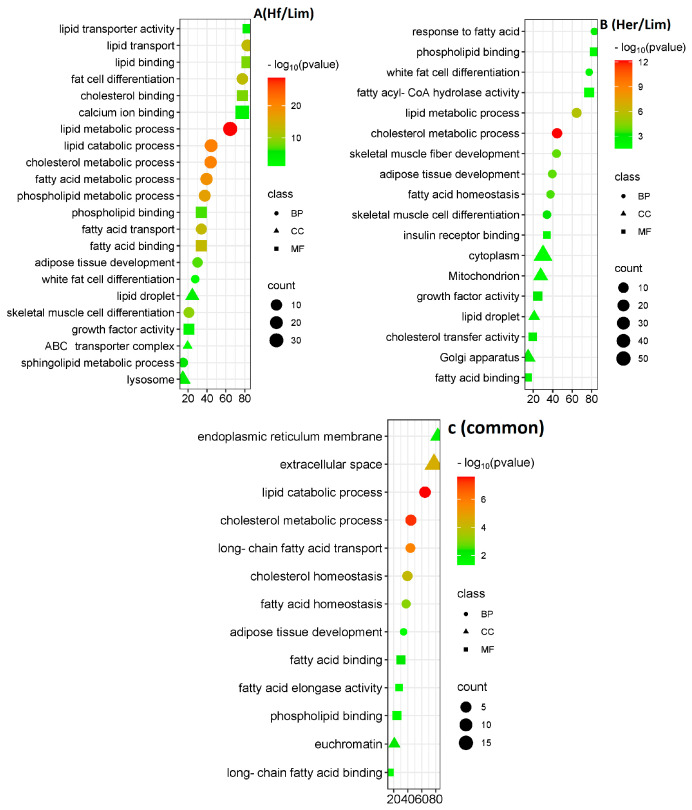
Functional gene ontology annotations for the differentially expressed genes. Bubble plots Her/Lim (**A**), Hf/Lim (**B**), and common (**C**) show the three independent GO information categories of biological processes, molecular functions, and cellular components, respectively. The color scale and the circle on the right-hand side illustrate the indicated pathways’ significance level and gene number. Larger bubbles signify more genes associated with a particular process, while smaller bubbles indicate fewer genes. All GO categories listed exhibit enrichment with *p* > 0.05. Hf—Holstein Friesian; Her—Hereford; Lim—Limousine.

Gene ontology (GO) provides a standardized framework for classifying gene functions globally. In this study, we utilized GO to understand the roles and functions of differentially expressed genes across the studied breeds, mapping them to three ontologies: molecular function (MF), cellular component (CC), and biological process (BP) (Figure 5; Supplementary Table S5).

Bubble plots for Hf/Lim (A), Her/Lim (B), and common (C) show the three independent GO categories. The color scale and the circles on the right side represent the pathways’ significance level and the number of associated genes. Larger bubbles signify more genes involved in a process, while smaller ones represent fewer. All listed GO categories showed significant enrichment with *p* < 0.05.

In the Her/Lim comparison, BP analysis showed DEGs enriched in processes, such as the lipid metabolic process, cholesterol metabolic process, skeletal muscle fiber development, adipose tissue development, fatty acid homeostasis, skeletal muscle cell differentiation, response to fatty acid, and white fat cell differentiation. CC analysis indicated that these DEGs were mainly associated with the lipid droplet, cytoplasm, Golgi apparatus, and mitochondrion. MF analysis highlighted functions such as growth factor activity, cholesterol transfer activity, fatty acid binding, insulin receptor binding, phospholipid binding, and fatty acyl-CoA hydrolase activity.

In the Hf/Lim comparison, BP terms included the lipid metabolic process, lipid catabolic process, cholesterol metabolic process, fatty acid metabolic process, phospholipid metabolic process, fatty acid transport, lipid transport, fat cell differentiation, skeletal muscle cell differentiation, adipose tissue development, sphingolipid metabolic process, and white fat cell differentiation. CC terms involved the cytoplasm, lipid droplet, ATP-binding cassette (ABC) transporter complex, and lysosome. MF terms encompassed fatty acid binding, lipid binding, cholesterol binding, phospholipid binding, lipid transporter activity, calcium ion binding, and growth factor activity.

For the common genes, BP analysis indicated enrichment in the lipid catabolic process, cholesterol metabolic process, long-chain fatty acid transport, cholesterol homeostasis, fatty acid homeostasis, and adipose tissue development. CC terms were mainly related to the endoplasmic reticulum membrane, extracellular space, and euchromatin. MF terms included fatty acid binding, long-chain fatty acid binding, fatty acid elongase activity, and phospholipid binding, suggesting a significant role of these DEGs in fat metabolism and energy regulation.

## 4. Discussion

In this study, we tried to elucidate the complex genetic mechanisms underlying IMF development in beef cattle, mainly focusing on the Holstein, Hereford, and Limousine breeds. We identified DEGs significantly associated with IMF deposition using RNA-Seq technology and advanced bioinformatics tools. These genes offer a deeper understanding of the biological processes and molecular functions of marbling, thereby providing potential genetic markers for selective breeding programs. Since this study’s main aim is to identify key and hub high-traffic genes affecting marbling, this transcriptome served as the foundation for conducting gene expression analyses related to the maturation of marbling in beef cattle. It has been proven that networks are dependable for representing genomic data [19]. Interpreting upregulated and downregulated genes regarding their topological aspects is imperative for extensive PPI networks. Consequently, it heavily relies on integrated local components, such as the distribution hub gene of the degree [20]. This parameter was employed to scrutinize the DEG dataset’s hub genes in separate PPI networks to infer the network significance (Figure 3). Analysis of the studied networks identified essential genes exhibiting notable expression, which can be recognized as biomarkers.

The 82 common genes identified between the Her/Lim and Hf/Lim comparisons were classified into four functional groups: adipogenesis, lipid metabolism, lipid storage, and skeletal muscle remodeling. This classification was based on GO terms, KEGG pathways, and the literature. Supplementary Table S3 provides detailed annotations, with selected genes discussed further below.

### 4.1. Common Genes Related to Adipogenesis

*SMAD3* was identified as the largest hub gene in the network, being downregulated in both Hf/Lim (log2FC = −0.60) and Her/Lim (log2FC = −0.61). Downregulation of *SMAD3* may reflect reduced inhibitory signaling, potentially correlating with higher IMF, though causality remains unconfirmed. Our results suggest that *SMAD3* may play a regulatory role in intramuscular fat deposition, although this requires experimental confirmation. As a key mediator in the TGFβ pathway, *SMAD3* regulates preadipocyte differentiation and represses myogenic differentiation [21]. TGFβ suppresses adipogenesis through *SMAD3* by interacting with C/EBPs, thereby inhibiting the transcription of the PPARG promoter [22]. Once activated, *SMAD3* forms complexes with *SMAD4*, which then move to the nucleus to activate or repress gene expression. Specifically, *SMAD3* binds to the basic helix–loop–helix domain of *MYOD*, preventing the formation of MYOD-E12/47 dimers [23]. These findings indicate that elevated SMAD3 levels can inhibit both muscle regulatory factors and adipogenesis via TGFβ–SMAD3 signaling, highlighting its dual suppressive role in muscle and fat cell differentiation [24]. The *SMAD3* gene is a negative regulator of muscle and adipose tissue development, and its downregulation in our analysis can suggest a positive impact on IMF development.

Catenin beta 1 (*CTNNB1*) was identified in our analysis as a gene within the shared network, showing upregulation in both Hf/Lim (2.07) and Her/Lim (1.12). While CTNNB1 is generally known to inhibit adipogenesis, in this context, *CTNNB1* upregulation may reflect tissue remodeling processes, with unclear implications for IMF deposition. *CTNNB1*, known for its role in apoptosis, may influence adipocyte differentiation indirectly through muscle tissue remodeling. It also triggers the MAPK kinase (MAPKK) cascade and the Wnt receptor signaling pathway through its gene product, β-catenin [25]. The Wnt signaling pathway can suppress adipocyte differentiation through mechanisms that are dependent or independent of β-catenin. In the Wnt/β-catenin pathway, β-catenin functions as a vital transcriptional coactivator [25].

*WNT1* is upregulated in Hf/Lim (3.44) and Her/Lim (2.12), and its increased expression in marbled muscle may reflect a complex transcriptional environment rather than directly influencing IMF levels. Wnt signaling through β-catenin plays a significant role in determining the fate of mesenchymal stem cells (MSCs) [26]. MSCs originating from the mesoderm are multipotent and can differentiate into various cell types, including adipocytes, osteocytes, chondrocytes, and myocytes [27]. Adipogenesis is the process by which MSCs develop into mature fat-storing adipocytes. While the Wnt pathway is also involved in myogenesis and chondrogenesis, its distinct regulatory effects on adipogenesis versus osteogenesis are well established [28]. Stabilization of β-catenin, either by the endogenous expression of canonical *WNT1* or a stable β-catenin mutant in multipotent progenitor cells, inhibits adipogenesis while promoting osteoblast formation [29]. Additionally, activating canonical Wnt signaling in preadipocytes through the enforced expression of *WNT1* or a dominant-stable form of β-catenin suppresses adipogenesis by blocking the expression of key transcription factors *PPARG* and *C/EBPα* [26].

### 4.2. Common Genes Related to Lipid Metabolism (Breakdown, Synthesis, and Regulation of Lipids)

*SCD*, the second hub gene in the shared network, was downregulated in Hf/Lim (−0.99) and Her/Lim (−0.98). Known for its role in lipid metabolism, this downregulation may reflect changes related to fat content but requires further validation. Lim et al. (2017) found a strong association between IMF content and *SCD* expression [30]. Other studies further support this link, showing that *SCD* is a key gene in adipocyte differentiation, catalyzing the production of monounsaturated fatty acids (MUFAs) [31]. The downregulation of *SCD* may indicate changes in lipid composition or a reduction in fatty acid synthesis in highly marbled muscles [32]. It catalyzes the Δ9-cis desaturation of fatty acyl-CoAs, producing two MUFAs, oleic acid (C18:1n9c) from stearate and palmitoleic acid (C16:1) from palmitate [33]. These fatty acids are major components of membrane phospholipids and triglycerides stored in adipocytes and adipose tissue [34]. As the main products of *SCD*, oleic and palmitoleic acids are the primary MUFAs found in fat depots and membrane phospholipids. Different *SCD* isoforms may be expressed between muscle and subcutaneous fat tissues [35].

*FGF10*, an upregulated hub gene in high-marbling breeds (Hf/Lim 1.56; Her/Lim 2.26), has been implicated in adipogenesis and myogenesis based on transcriptomic data. *FGF10* may contribute to lipid metabolism, but its exact role in IMF remains unclear [36]. The *FGF10* gene plays crucial roles in adipocyte metabolism and development, mediating biological responses by activating FGF receptor 2b (*FGFR2B*) with heparin–heparan sulfate in a paracrine manner. The *FGF10* gene is significant in adipogenesis, partly by contributing to the expression of C/EBPb through an autocrine–paracrine mechanism [37]. Furthermore, *FGF10* stimulates preadipocyte proliferation through the Ras-MAPK pathway, followed by cyclin D2-dependent phosphorylation of p130. It also induces adipogenesis by promoting the expression of *pRb* through the Ras-MAPK pathway, leading to the binding of *pRb* with *C/EBPa* [38]. Therefore, exploring *FGF10* genetic polymorphisms may aid breed improvement strategies for body measurement and carcass quality traits in beef cattle. The provided evidence indicates that *FGF10* plays a crucial role in lipid metabolism.

Retinoid X Receptor Alpha (*RXRA*), a nuclear receptor involved in fatty acid metabolism regulation, shows differential expression in Hf/Lim (0.81) and Her/Lim (2.58) and interacts with multiple genes in the network. While *RXRA* is known to modulate lipid metabolic pathways in muscle tissue, the observed expression patterns may reflect associated metabolic activity rather than directly drive IMF deposition in this context. *RXRA* may be involved in lipid regulation via PPARG interaction, though its role in IMF is indirect [39]. It is widely recognized as one of the most extensively distributed transcriptional regulators and is deeply involved in controlling genes crucial for lipid metabolism [40]. The *RXRA* gene is implicated in fat metabolism through its *PPARG* induction, which regulates triglyceride synthesis and fatty acid oxidation [41]. Collectively, these findings suggest a possible role for RXRA in lipid metabolism during marbling development, but further experimental validation is required to confirm its direct impact.

Squalene epoxidase (*SQLE*), a product of a marbling-related gene, is upregulated in Hf/Lim (1.56) and Her/Lim (2.00). According to DAVID data, *SQLE* plays a role in cholesterol metabolism and steroid biosynthesis. This enzyme, involved in cholesterol production, is essential for converting squalene to 2,3-oxidosqualene, a critical step in the early stages of cholesterol synthesis [42]. *SQLE* has been identified as a differentially expressed gene in both cattle and pigs, potentially associated with fat deposition and meat quality traits. Although its exact role in muscle development remains unclear, insights from model organisms (e.g., mice and pigs) suggest it may have a similar function in bovine intramuscular fat regulation. However, species-specific mechanisms should be considered [43]. These findings suggest that bovine *SQLE* might influence IMF through lipid biosynthesis, but its role is speculative [43].

*PRKCA* (Hf/Lim −0.94; Her/Lim −0.56) was identified as a marbling-related gene. PRKCA is part of a group of serine- and threonine-specific protein kinases that can be activated by calcium and the second messenger, diacylglycerol [44]. This gene is involved in various cellular processes, such as cell adhesion, cell transformation, cell cycle checkpoints, and cell volume control [44]. *PRKCA* may be engaged in IMF-related signaling, though further evidence is needed [45]. The functions of *PRKCA* include negative modulation of the insulin receptor signaling pathway and regulation of muscle contraction. Insulin sensitivity is crucial for IMF accumulation, leading to preadipocyte proliferation and enhancing muscle glucose oxidation [46]. While known to inhibit apoptosis and enhance preadipocyte proliferation in other systems, its specific role in IMF accumulation here requires further validation.

*HMGCS2*, another discussed gene, is upregulated in Hf/Lim (3.70) and Her/Lim (2.04). Previous studies have shown that *HMGCS2* promotes fatty acid α-oxidation and ketone production in hepatoma cells, playing a vital role in fatty acid oxidation and overall lipid metabolism. Its upregulation suggests possible shifts in fat metabolism, pending functional validation. Moreover, the *ANKRD2* gene may improve meat quality traits by regulating the expression of genes, such as *HMGCS2* and *GK2*, which are involved in glucose and lipid metabolism, thereby influencing intracellular fat levels and fatty acid composition [47].

### 4.3. Common Genes Related to Lipid Storage (Lipid Accumulation and Fat Droplet Formation)

Perilipin 2 (*PLIN2*) is the third hub gene in the common network and is downregulated in Hf/Lim (−1.20) and Her/Lim (−1.85). *PLIN2*, a marker of lipid droplets, protects them from lipolysis and promotes fatty acid storage as triglycerides. *PLIN2*′s downregulation may be associated with altered lipid droplet dynamics, potentially favoring increased fat turnover [48]. Previous research revealed that a polymorphism in the porcine *PLIN2* gene was linked to higher carcass lean content in Italian Duroc pigs, with elevated *PLIN2* mRNA expression in the skeletal muscle of pigs with increased intermuscular fat, suggesting a role in lipid accumulation in muscle [49]. Ryan et al. (2022) demonstrated that lipid accumulation results in the formation of LDs, organelles responsible for lipid storage and use [50]. LDs consist of a neutral lipid core surrounded by a phospholipid monolayer and various proteins, with perilipin (PLIN) being the most abundant and vital for regulating lipid storage by shielding LDs from lipase activity [51]. *PLIN2* is expressed throughout the body [52], and its overexpression in the heart has been linked to significant lipid accumulation in the myocardium, suggesting its role in LD stabilization [53]. In hepatocytes, *PLIN2* overexpression has been shown to protect against autophagy, while its downregulation promotes triglyceride (TG) breakdown via autophagy [53]. In vitro studies have also revealed that *PLIN2* deficiency reduces lipid accumulation by weakening the LD-associated lipase barrier, leading to increased lipolysis [54]. Given its role in shielding LDs from lipolysis, its reduced expression suggests a potential increase in fat turnover, which may influence IMF accumulation.

Apo lipoprotein E (*APOE*) is downregulated in Hf/Lim (−1.13) and Her/Lim (−1.08). It is not one of the hub proteins of the common network, but it plays a vital role in lipid metabolism. *APOE* is a structural component of all lipoprotein particles except LDL, acting as a high-affinity ligand for lipoprotein receptors [55]. Research has also highlighted the significant role of *APOE* carried by circulating triglyceride-rich lipoproteins (TGRL) in adipocyte metabolism, with studies showing that endogenously expressed *APOE* can notably affect triglyceride and fatty acid metabolism in adipocytes in vitro [56]. *APOE* plays a critical role in lipid transport and storage, with its downregulation potentially indicating reduced fat mobilization and increased fat storage in muscle tissue.

The gene of fatty acid binding protein 4 (*FABP4*) plays a role in fatty acid metabolism and is upregulated in Hf/Lim (2.95) and Her/Lim (2.75). It is predominantly expressed in adipocytes and encodes proteins involved in fatty acid uptake, transport, and metabolism [57]. *FABP4* is strongly associated with fat deposition through its involvement in long-chain fatty acid transport and interorgan nutrient partitioning during development. Increased *FABP4* levels correlate with adipocyte content in muscle tissue, suggesting its potential as a marker for intramuscular adipogenesis [58]. Research has shown that *FABP4* genotypes influence the marbling score, subcutaneous fat depth, and fatty acid composition in IMF, particularly in Japanese Black cattle [59] and the Wagyu × Limousine F2 population [60]. The gene is located in genomic regions associated with quantitative trait loci (QTLs) for marbling in *Bos taurus* cattle [61], IMF in various breeds like Angus, Hereford, Brahman, and Santa Gertrudis [62], and carcass traits in Korean cattle [62]. Polymorphisms in *FABP4* have been significantly linked to marbling and IMF content [60]. In pigs, *FABP4* is considered a candidate gene for fatness traits, with its gene located in QTL regions for traits like back fat thickness and IMF content [63]. The expression of *FABP3* and *FABP4* proteins has been correlated with triacylglycerol content and marbling in cattle [64]. In chickens, single-nucleotide polymorphisms (SNPs) in *FABP3* and *FABP4* genes have been associated with IMF content [65] and overexpression of *FABP4* in chicken adipocytes, significantly increasing lipid accumulation [66]. The data mentioned above suggests *FABP4* is a key regulator of IMF accumulation, with its expression potentially modulating lipid distribution among muscle fibers.

Another identified common gene, *ELOVL3*, is upregulated in Hf/Lim (4.70) and Her/Lim (3.14) breeds. *ELOVL3* is an elongase that belongs to the elongation of the very long-chain fatty acids protein family (ELOVLs) [67]. It was first identified due to its upregulated expression in the brown adipose tissue of mice in response to cold stress [68]. *ELOVL3* is also highly expressed in white adipose tissue, sebaceous glands of the skin, and the liver [69]. It plays a key role in regulating the synthesis of endogenous saturated, very long-chain fatty acids and triglycerides. Research has shown that *ELOVL3* helps replenish the intracellular pool of triacylglycerols, which is crucial for maintaining lipid homeostasis [38]. Additionally, *ELOVL3* expression has increased significantly during the differentiation of bovine skeletal muscle-derived satellite cells (MDSCs), suggesting a role in muscle development [70]. The strong upregulation of *ELOVL3* in high-marbling breeds supports its involvement in muscle lipid metabolism and IMF deposition, as seen in previous studies on adipose tissue differentiation.

### 4.4. Common Genes Related to Skeletal Muscle Remodeling During IMF Deposition

Skeletal muscle remodeling during increased IMF deposition involves changes in muscle structure and function [71]. Identified DEGs are involved in extracellular matrix (ECM) remodeling, inflammation, and signaling pathways that can affect muscle integrity, repair, and remodeling, as shown in Supplementary Table S3. Some of them are briefly discussed below.

The extracellular matrix remodeling-related gene *COL3A1* is downregulated in a common set of DEGs (Hf/Lim −3.27, Her/Lim −0.77). It encodes type III collagen, which maintains the structural integrity of tissues. Reduced expression might be associated with muscle stiffness and flexibility changes during fat infiltration [72]. Another DEG, which encodes type IX collagen *COL9A1*, is upregulated in Hf/Lim (3.59) and Her/Lim (2.58). It is essential for maintaining the ECM and cartilage [73]. Its upregulation suggests that collagen production is modulated during muscle remodeling as fat infiltrates the tissue. Downregulated ADAM metallopeptidase with thrombospondin type 1 motif 4 *ADAMTS4* (Hf/Lim −2.40, Her/Lim −2.56) is involved in the breakdown of aggrecan and other ECM components [74]. Its reduced activity could lead to a more stable ECM structure, supporting tissue remodeling and fat deposition in skeletal muscle.

The anti-inflammatory cytokine that regulates muscle repair and ECM remodeling during increased IMF deposition, *IL10*, is upregulated in high-marbling cattle (Hf/Lim 2.89, Her/Lim 2.01). It may help muscles recover and adapt to tissue remodeling [75]. Moreover, the pro-inflammatory cytokine *IL17A* is involved in the immune response. In our study, it is downregulated and common for both examined comparisons (Hf/Lim −2.98, Her/Lim −4.51). Its downregulation could reduce inflammation during muscle remodeling, allowing more efficient muscle adaptation to fat deposition [76]. Growth differentiation factor 15 (*GDF15*; Hf/Lim 3.81, Her/Lim 2.44) regulates inflammation and muscle cell stress responses, possibly contributing to muscle adaptation during fat infiltration [77].

Some of the identified genes are involved in energy metabolism and muscle adaptation. Nuclear receptor subfamily 1 group H member 4 (*NR1H4*) (Hf/Lim 3.14, Her/Lim 2.01), also known as farnesoid X receptor (*FXR*), regulates bile acid and lipid metabolism but can also modulate metabolic processes in muscle cells, which may impact muscle during adaptation to increased fat deposition [78]. Melanocortin 4 receptor (*MC4R*) (Hf/Lim 4.18, Her/Lim 3.01) is involved in energy homeostasis and potentially contributes to muscle adaptation during IMF deposition [79]. Growth differentiation factor 3 (*GDF3*) (Hf/Lim 4.62, Her/Lim 3.16) is involved in energy balance, adipogenesis, and muscle regeneration. It is an obesity-induced regulator of TGFβ, which may impact muscle metabolism and tissue remodeling processes [80].

Finally, some muscle contraction and structure-related genes were also identified as DEGs. Skeletal muscle actin alpha 1 (*ACTA1*) (Hf/Lim 1.26, Her/Lim 2.78) is essential for muscle contraction. The increased expression could indicate compensatory muscle growth or structural changes during IMF deposition [81]. Pericentric (*PCNT*; Hf/Lim 1.63, Her/Lim 1.01) is involved in cell division and the organization of the cytoskeleton [82], which could play a role in muscle cell adaptation during remodeling. The indicated genes showed a significant change in expression in high-marbling Hf/Her muscle tissue compared to low-marbling Lim muscle tissue. These results show that the genes in the common network may help the muscle tissue adapt to the growing presence of IMF, affecting muscle integrity, regeneration, and structure.

### 4.5. Common Signaling Pathways

We conducted KEGG pathway analyses to determine pathways in relation to the DEGs using the DAVID online tool. This study employed biological pathway analysis to explore the biological importance or functional correlation among the notable genes linked to the marbling score. Some known signaling mechanisms regulating IMF deposition, such as Wnt, PPAR, and PI3K–Akt, were discussed.

The signaling pathway plot reveals that five DEGs associated with the Wnt signaling pathway are *SMAD3*, *WNT1*,* CTNNB1*,* NFATC3*, and *PRKCA*. The Wnt signaling pathway can affect IMF content by modulating several adipocyte metabolic pathways [83].

Wnt signaling pathway involvement in adipogenesis/IMF deposition was described above when the DEGs *CTNNB1* and *WNT1* were discussed.

The Wnt signaling pathway promotes myogenic and osteogenic differentiation while inhibiting adipogenesis by suppressing key adipogenic regulators, such as PPARG and C/EBPα. This dual role is critical during embryonic development and contributes to reduced fat accumulation and obesity risk by limiting the differentiation of mesenchymal stem cells into adipocytes [84].

Once optimal muscle growth is achieved, inhibiting the Wnt signaling pathway might be beneficial to boost fat cell production [85].

In this study, six genes (*HMGCS2*, *ACOX1*, *FABP4*, *PLIN2*, *RXRA*, and *SCD*) involved in the peroxisome proliferator-activated receptor (PPAR) signaling pathway were linked to meat quality. These genes are recognized as critical regulators within the PPAR pathway, which is associated with adipogenesis and marbling traits in beef cattle. Specifically, their expression in muscle has been shown to correlate positively with marbling [86]. Among these, three genes exhibited higher expression, while the others showed lower expression levels. Previous studies have also highlighted the role of *HMGCS2* [87], *SCD* [30], *ACOX1* [88], *FABP4* [64], *PLIN2* [89], and *RXRA* [90] in fat metabolism and IMF content, supporting the current findings. These genes are, therefore, important for understanding the factors influencing meat quality in livestock. The PPAR signaling pathway, known for its role in lipid metabolism, is a crucial biological mechanism influencing meat quality in mammals [41]. Peroxisome proliferator-activated receptor gamma, the primary transcription regulator of this pathway, plays a key role in controlling the transcription of genes involved in adipogenesis-related processes [91]. In cattle, the PPAR pathway activates various downstream pathways, such as those governing lipid metabolism, adipocyte differentiation, and gluconeogenesis [41]. PPARs are nuclear hormone receptors activated by fatty acids and their derivatives, primarily involved in regulating lipid metabolism, adipocyte differentiation, gluconeogenesis, and thermogenesis by modulating multiple target genes. There are three *PPAR* subtypes (*PPARA*, *PPARB/D*, and *PPARG*), each with distinct expression patterns. *PPARA*, located on chromosome 5 near a QTL for traits like backfat thickness and feed intake, regulates genes involved in lipid metabolism [92]. *PPARG*, located on chromosome 13 near a QTL for body weight, promotes adipocyte differentiation and fat deposition and regulates lipid metabolism and glucose homeostasis [93].

**Figure 6:**
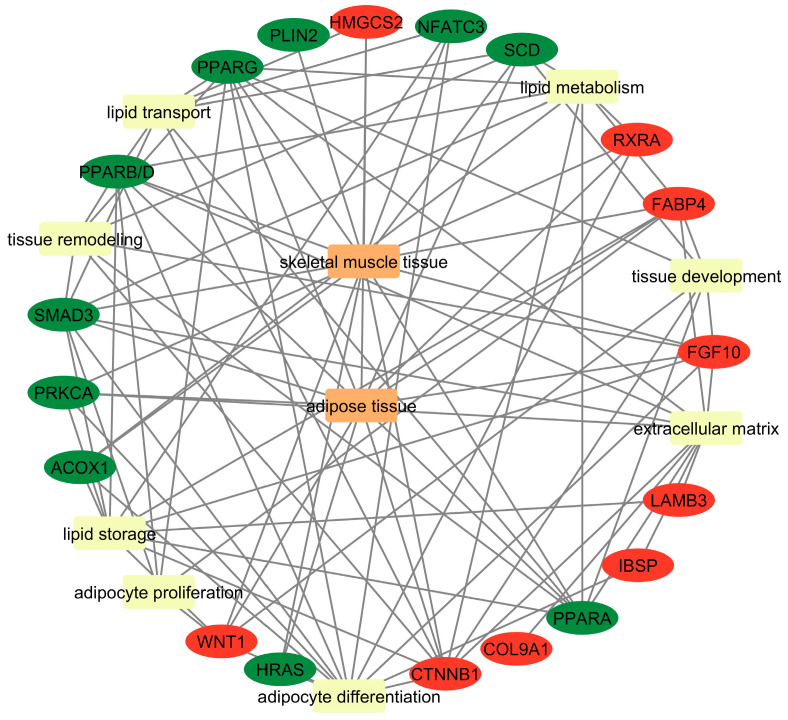
The network of dependencies between identified common DEGs and biological processes related to breed-independent intramuscular fat deposition. Green color—a lower expression of DEGs; red—a higher expression of DEGs; yellow—biological process; orange—tissue.

NGS analysis aimed at discovering biological pathways related to marbling revealed that the PI3K–Akt pathway in the common breeds is significant for IMF development. Genes identified in this pathway include *HRAS*, *COL9A1*, *FGF10*, *IBSP*, *LAMB3*, *PRKCA*, and *RXRA*. The PI3K–Akt pathway is critical in regulating adipogenesis, including preadipocyte differentiation and the transformation of brown and white fat cells [94]. Activated by insulin and other growth factors, this pathway promotes lipid synthesis and storage [38]. Insulin signaling via the PI3K–Akt pathway activates transcription factors, such as PPARG, which is crucial for adipocyte differentiation [95]. Moreover, inhibiting PI3K/Akt activation can suppress adipogenesis and hinder the differentiation of preadipocytes [96]. The pathways mentioned above and genes may be involved in adipocyte differentiation, lipid metabolism, lipid storage, and skeletal muscle remodeling during IMF deposition. Accordingly, the DEGs identified in this study may represent promising targets for genetic improvement in marbling traits. A brief summary of the discussion is presented in Table 2, where DEGs are divided into core and contextual genes, and in Figure 6.

While this study provides valuable transcriptomic insights into IMF deposition in cattle, some limitations should be acknowledged. The absence of functional validation limits the ability to confirm the biological relevance of the identified genes and pathways. Additionally, the reliance on pathway enrichment and PPI network analyses, which are based on existing databases, may not fully capture breed-specific regulatory mechanisms. Future studies should include functional validation (e.g., in vivo *or* in vitro experiments), breed-specific analyses, and multi-omics approaches (e.g., proteomics, metabolomics) to better understand the genetic and molecular basis of IMF deposition. Incorporating non-genetic factors, such as diet, age, and management practices, will provide a more holistic view of IMF development. Addressing these limitations will enhance the reliability of the findings and support the development of robust genetic markers for improving beef quality through selective breeding.

## 5. Conclusions

This study identified key genes and pathways associated with intramuscular fat deposition and muscle development in cattle, revealing complex regulatory networks influencing marbling. Further validation and additional analyses, including multi-omics approaches and gene–environment interaction studies, are necessary for the practical application of these findings. Moreover, considering non-genetic factors, such as diet and management practices, will provide a more comprehensive understanding of the fat deposition processes. These efforts can contribute to developing more precise genetic markers and improving beef quality through selective breeding.

## Figures and Tables

**Table 1:** Summary of bovine genome read mapping.

Breed	Total Reads	Mapped	Paired Seq	Read1	Read2	Properly Paired	Mate-Mapped	Singletons
Lim1	129.8 M	123.5 M	104.4 M	52.2 M	52.2 M	94.5 M	95.2 M	2.91 M
Lim2	120.9 M	113.2 M	94.4 M	47.2 M	47.2 M	82.5 M	83.4 M	3.34 M
Lim3	115.4 M	108.1 M	91.2 M	45.6 M	45.6 M	80.3 M	81.1 M	2.80 M
Lim4	122.5 M	114.5 M	95.9 M	47.9 M	47.9 M	84.0 M	84.7 M	3.23 M
Hf1	118.3 M	94.6 M	92.9 M	46.5 M	46.5 M	46.1 M	65.7 M	3.49 M
Hf2	337.2 M	312.6 M	268.3 M	134.1 M	134.1 M	232.9 M	235.0 M	8.61 M
Hf3	135.0 M	122.6 M	109.0 M	54.5 M	54.5 M	91.4 M	92.7 M	3.97 M
Hf4	151.5 M	145.6 M	128.0 M	64.0 M	64.0 M	88.6 M	90.5 M	11.0 M
Her1	143.3 M	139.3 M	112.6 M	6.0 M	59.8 M	98.5 M	103.5 M	3.65 M
Her2	196.4 M	187.2 M	146.2 M	73.1 M	73.1 M	131.9 M	133.0 M	3.89 M
Her3	174.2 M	166.1 M	135.1 M	63.1 M	45.1 M	112.2 M	113.2 M	4.56 M
Her4	131.3 M	126.0 M	104.2 M	52.1 M	52.1 M	85.2 M	86.4 M	3.64 M

**Table 2:** Core and contextual DEGs identified in this study, and their putative functions in IMF-related biological processes.

**Core Genes**	**Brief Description of Function**	**Reference**
*SMAD3*	Regulates adipogenesis and myogenesis via the TGFβ–SMAD pathway; negative regulator of adipogenesis	[21,22,23,24]
*FABP4*	Transports long-chain fatty acids; IMF marker; QTL in many breeds	[57,58,59,60,61,62,63,64,65,66]
*RXRA*	Nuclear receptor; regulates lipid pathways (with PPARG); influences fat metabolism; known lipid regulator	[39,40,41,90]
*SCD*	Enzyme synthesizing MUFA; IMF and meat quality marker	[30,31,32,33,34,35]
*PLIN2*	Protects lipid droplets from lipolysis; regulates fat storage	[48,49,50,51,52,53,54,89]
*FGF10*	Regulates adipogenesis and preadipocyte proliferation via MAPK and pRb–C/EBP	[36,37,38]
*ELOVL3*	Biosynthesis of long-chain fatty acids; activated in adipogenesis	[38,67,68,69,70]
*WNT1*	Blocks adipogenesis; activates Wnt/β-catenin; influences MSC fate	[26,27,28,29]
*CTNNB1*	Transcriptional activator in Wnt; muscle tissue remodeling	[25]
**Contextual Genes**	**Brief Description of Function**	**Reference**
*SQLE*, *HMGCS2*, *APOE*, *NR1H4*, *ACOX1*	Involved in lipid biosynthesis, transport, oxidation, and adaptation	[42,43,47,55,56,78,87,88]
*PRKCA, MC4R*	Modulate insulin signaling and energy homeostasis	[44,45,46,79]
*COL3A1*, *COL9A1*, *ADAMTS4*, *PCNT*, *ACTA1*	Extracellular matrix remodeling and cytoskeletal reorganization	[72,73,74,81,82]
*IL10*, *IL17A*, *GDF15*, *GDF3*	Inflammatory response and immune-mediated tissue remodeling	[75,76,77,80]
*NFATC3*, *HRAS*, *IBSP*, *LAMB3*	Participation in key signaling pathways regulating muscle and fat cell biology (Wnt, PI3K–Akt, PPAR)	[83,88,94]

## Data Availability

The datasets generated during the current study are available in the NCBI Sequence Read Archive (SRA) repository: https://www.ncbi.nlm.nih.gov/bioproject/PRJNA1187860/; analysis data are included in this published article and its supplementary information files. Information on the data acquired in this project is available upon request from the corresponding author, tomasz_sadkowski@sggw.edu.pl.

## Supplementary Materials

The following supporting information can be downloaded at https://www.mdpi.com/article/10.3390/genes16080984/s1, Table S1: The most critical genes regulating the gene network with a high degree in Her/Lim and Hf/Lim breeds; Table S2: The most critical genes regulating the gene network with a high degree of a common network; Table S3: Common 82 genes identified between Hf/Lim and Her/Lim breeds, which most affect marbling between the two study groups; Table S4: Signaling pathways of differentially expressed genes in Her/Lim, Hf/Lim, and common breeds; Table S5: Gene ontology of differentially expressed genes in Her/Lim, Hf/Lim, and common breeds.
