# Supplementary material for: Unveiling Conserved Molecular Pathways of Intramuscular Fat Deposition and Shared Metabolic Processes in Semitendinosus Muscle of Hereford, Holstein, and Limousine Cattle via RNA-Seq Analysis
Source: Genes (Basel). 2025 Aug 21;16(8):984. doi: 10.3390/genes16080984 (PMC12385640; doi:10.3390/genes16080984)
Supplement: Supplementary file 1 [file genes-16-00984-s001.zip › genes-3716074-supplementary.pdf]

Table S1. The most critical genes regulating the gene network with a high degree in Her/Lim and Hf/Lim breeds.

Gene Symbol	Group	P-value	Log2Fold Change	Degree	Biological Process
**MYOD1**	Hf/Lim	2.25E-03	-0.9148	59	muscle organ development, skeletal muscle tissue development
**TREM1**	Hf/Lim	8.84E-03	2.724331	59	brown fat cell differentiation
**TBX21**	Hf/Lim	3.48E-03	2.113158	57	cell fate specification
**SCARB2**	Hf/Lim	2.038E-09	-1.57153	57	aminophospholipid transport
**GPAM**	Hf/Lim	6.61E-03	1.326119	49	intestinal lipid catabolic process, phospholipid catabolic process
**VSNL1**	Hf/Lim	9.32E-03	2.521835	58	negative regulation of MAP kinase activity
**PNLIP**	Hf/Lim	6.65E-03	2.396929	49	lipid metabolic process, lipid catabolic process, intestinal cholesterol absorption,
**FOXO1**	Hf/Lim	1.39E-03	1.794924	56	negative regulation of fat cell differentiation, response to fatty acid
**TRAF3**	Hf/Lim	4.08E-02	0.919475	58	Wnt signaling pathway
**DGAT1**	Hf/Lim	1.32E-02	0.504089	54	long-chain fatty-acyl-CoA metabolic process, fatty acid homeostasis, lipid storage
**SMAD3**	Her/Lim	3.14E-03	-0.61732	59	regulation of insulin secretion, positive regulation of MAPK cascade
**IL6**	Her/Lim	4.23E-03	-1.95373	58, 30	negative regulation of fat cell differentiation
**SCD**	Her/Lim, common	1.51E-03	-0.98243	56, 27	fatty acid biosynthetic process, unsaturated fatty acid biosynthetic process, response to fatty acid
**SLC2A4**	Her/Lim	6.62E-03	-0.68875	53	brown fat cell differentiation
**PLIN2**	Her/Lim, common	2.75E-10	-1.85421	53, 25	long-chain fatty acid transport, lipid storage, lipid particle disassembly
**SREBF1**	Her/Lim	8.11E-03	-0.63675	51	regulation of the fatty acid metabolic process, fat cell differentiation
**RXRA**	Her/Lim, common	2.22E-05	0.80683	49, 21	fat cell differentiation
**NR3C1**	Her/Lim	4.75E-03	-0.74951	47	gene expression

**XBP1**	Her/Lim	8.35E-05	0.991080241	47	muscle organ development, cholesterol homeostasis
**PNPLA2**	Her/Lim	1.85E-07	-1.37106	46	lipid catabolic process, lipid storage, lipid homeostasis

Table S2. The most critical genes regulating the gene network with a high degree of common network

Gene Symbol	Group	Log2 Fold Change (Her/Lim)	P-value (Her/Lim)	Log2 Fold Change (Hf/Lim)	P-value (Hf/Lim)	Degree	Biological Process
**SMAD3**	common	-0.61732	1.25E-02	-0.607052	6.61E-05	29	cholesterol metabolic process, steroid biosynthesis
**SCD**	common	-0.98243	1.34E-06	-0.99857	2.11E-03	27	cell fate specification, positive regulation of skeletal muscle cell proliferation
**PLIN2**	common	-1.85421	3.73E-02	-1.20599	2.42E-03	24	regulation of blood pressure
**SHH**	common	2.21815	1.78E-05	3.559631	3.08E-02	23	cholesterol homeostasis
**SQLE**	common	2.0023	1.41E-03	1.564019	2.25E-03	23	apoptotic process, positive regulation of lipopolysaccharide-mediated signaling pathway
**RXRA**	common	2.5852	2.54E-03	0.806829724	3.66E-03	20	hydrogen peroxide catabolic process
**NPPA**	common	2.283403	4.77E-02	3.530006	3.38E-02	18	cellular response to hepatocyte growth factor stimulus
**NR1H4**	common	2.012618191	1.91E-02	3.143756861	1.01E-05	17	positive regulation of gene expression
**PRKCA**	common	-0.56473	2.65E-02	-0.94462	4.63E-02	16	negative regulation of transforming growth factor beta

							receptor signaling pathway, negative regulation of BMP signaling pathway
**IL10**	common	2.013229	4.11E-02	2.89873	2.5E-03	16	positive regulation of the apoptotic process

Table S3. Common 82 genes identified between Hf/Lim and Her/Lim breeds, which most affect marbling between the two study groups

Gene symbol	Log2FoldChange (Her/Lim)	Log2FoldChange (Hf/Lim)	Biological Processes
**PLIN2**	-1.85421	-1.20599	long-chain fatty acid transport, lipid storage
**PRKCA**	-0.56473	-0.94462	apoptotic process, positive regulation of lipopolysaccharide-mediated signaling pathway
**SCD**	-0.98243	-0.99857	response to fatty acid, monounsaturated fatty acid biosynthetic process
**PNPLA1**	2.332502	3.819828	triglyceride catabolic process, lipid homeostasis
**SMAD3**	-0.61732	-0.607052	positive regulation of gene expression, BMP signaling pathway,
**APOE**	-1.08502	-1.13196	cholesterol catabolic process, lipid transport
**LIPK**	2.282991	3.047568	lipid catabolic process,
**GPX1**	-0.83905	-1.33758	hydrogen peroxide catabolic process
**NFE2L1**	-0.73465	-2.1385	cholesterol metabolic process
**ACOX1**	-0.78784	-0.99998	lipid metabolic process, long-chain fatty acid metabolic process
**COL3A1**	-0.77766	-3.27602	transforming growth factor beta1 production, positive regulation of Rho protein signal transduction
**ABCD1**	-0.71785	-1.10788	fatty acid beta-oxidation, fatty acid homeostasis
**PEDS1**	-0.64699	-0.87799	fatty acid metabolic process
**NAPEPLD**	-0.6329	-1.32165	lipid catabolic process
**NFATC3**	-0.79297	-0.794809703	regulation of transcription by RNA polymerase II
**IL13RA1**	-0.6097	-1.27661	cytokine-mediated signaling pathway
**IL17A**	-2.983657	-4.509115	Notch signaling pathway, gene expression,
**IL36G**	4.485926	5.639039	cellular response to lipopolysaccharide,
**TMEM64**	-0.57392	-0.94889	positive regulation of fat cell differentiation
**ECHDC3**	-0.58587	-1.31772	positive regulation of cellular response to insulin stimulus
**HRAS**	-0.5676	-0.95858	insulin receptor signaling pathway, adipose tissue development
**PTGR3**	-0.58816	-0.85855	negative regulation of fat cell differentiation
**THOP1**	-0.53536	-0.99414	peptide metabolic process

**ADAMTS4**	-2.5625	-2.3931	extracellular matrix organization
**AGPAT2**	-0.51687	-1.0041	phosphatidic acid biosynthetic process
**CBR4**	-0.57896	-1.10379	fatty acid biosynthetic process
**PGRMC2**	-0.52718	-1.34344	adipose tissue development
**FDX1**	-0.52972	-0.85263	cholesterol metabolic process
**ACOT8**	-0.81548	-0.82957	fatty acid catabolic process
**PLA2G12B**	2.106636	2.794826	phospholipid metabolic process, lipid catabolic process
**CYP51A**	2.55023	1.380266	positive regulation of Wnt signaling pathway
**RXRA**	2.5854	0.806829724	cell differentiation
**LAMB3**	1.060800008	0.896350902	brown fat cell differentiation
**ACTA1**	2.782534589	1.26783256	skeletal muscle thin filament assembly
**NPPA**	2.283403	3.530006	regulation of blood pressure
**ELOVL4**	2.00192	3.318786	fatty acid elongation, polyunsaturated fatty acid
**PPARγ**	3.0025	3.79685	positive regulation of Wnt signaling pathway
**SQLE**	2.0023	1.564019	cholesterol metabolic process
**PCNT**	1.01756	1.63575	positive regulation of intracellular protein transport, signal transduction
**IL10**	2.013229	2.89873	cellular response to hepatocyte growth factor stimulus,
**CEBPα**	4.01231	3.05458	positive regulation of MAP kinase activity
**DCT**	2.045562	3.207851	cell development
**HMGCS2**	2.046424	3.70966	acetyl-CoA metabolic process, cholesterol biosynthetic process
**ESRRG**	2.085379	1.842997	regulation of DNA-templated transcription
**PLA2G2F**	2.642001	3.622988	phospholipid metabolic process, lipid catabolic process
**CYP27B1**	2.110806	3.044765	cholesterol metabolic process
**TPD52L1**	2.827481	3.588829	positive regulation of MAP kinase activity
**CTNNB1**	1.12158	2.07289	positive regulation of apoptotic process
**PLD6**	2.770703	4.168581	lipid catabolic process
**SHH**	2.21815	3.559631	cell fate specification, positive regulation of skeletal muscle cell proliferation
**WNT1**	2.122992	3.440085	negative regulation of transforming growth factor beta receptor signaling pathway, negative regulation of BMP signaling pathway
**BTNL2**	2.18284	2.839216	positive regulation of MAP kinase activity
**ARRDC3**	2.193968	1.74606	protein transport
**GIP**	2.267395	3.969293	positive regulation of insulin secretion
**FGF10**	2.26948	1.560274	white fat cell differentiation
**MC4R**	3.013256	4.188523	regulation of metabolic process, insulin secretion
**NR1H4**	2.012618	3.143757	cholesterol homeostasis
**CYP11B1**	2.320485	3.5499	cholesterol metabolic process
**POU4F2**	2.008521	3.478913	negative regulation of adipose tissue development
**SP1**	3.33236	3.88548	positive regulation of gene expression
**ATF3**	2.337718	3.649084	skeletal muscle cell differentiation
**ADRB2**	1.39245	1.71981747	positive regulation of MAPK cascade

**ITIH4**	0.774106005	0.961716641	negative regulation of insulin secretion
**GDF15**	2.443727	3.810923	positive regulation of fatty acid oxidation,
**APOA4**	2.453227	3.698433	cholesterol biosynthetic process, response to lipid hydroperoxide
**SSTR5**	2.367411	3.407989	regulation of insulin secretion
**COL9A1**	2.58235	3.595921	extracellular matrix organization
**PLCXD3**	2.139248	3.236364	lipid metabolic process, signal transduction, lipid catabolic process
**FOXH1**	2.672555	3.499784	negative regulation of insulin secretion
**FABP4**	2.75156	2.95547	negative regulation of protein kinase activity, fatty acid metabolic process
**SLC46A1**	2.77245	3.77245	folic acid transport
**FABP9**	2.900461	5.163994	fatty acid transport, long-chain fatty acid transport
**CLPSL2**	2.934318	4.155318	lipid catabolic process
**MVD**	-0.66079	-0.80566	cholesterol biosynthetic process
**ELOVL3**	3.144699	4.700012	fatty acid elongation, saturated fatty acid
**GDF3**	3.165481	4.62482	positive regulation of fat cell differentiation
**IBSP**	3.321359	2.66265	cellular response to growth factor stimulus,
**GCG**	3.343344	5.566952	lipid biosynthetic process, protein kinase A signaling, negative regulation of apoptotic process
**ACVR1C**	3.818437	4.858696	lipid storage, insulin secretion
**IFN-TAU**	3.993281	6.104199	cellular response to growth factor stimulus
**IFNT3**	4.210628	5.055295	cellular response to growth factor stimulus
**IL5RA**	2.434219	3.679643	regulation of interleukin-5 production

Table S4. Signaling Pathways of differentially expressed genes in Her/Lim, Hf/Lim, and common breeds

Group	Signaling Pathway	Count	P-value	GENE
Her/Lim	MAPK signaling pathway	12	1.3E-05	HRAS, TAB1, CACNA1S, CSF1, ERBB2, IGF1R, MAPK3, MYOD1, PDGFA, PRKCA, STK3, DGAT1
Her/Lim	Cholesterol metabolism	8	3.6E-03	VAPA, APOA4, APOE, CYP27A1, NCEH1, OSBPL5, PLTP, SORT1
Her/Lim	Fatty acid metabolism	6	2.7E-03	ELOVL3, ELOVL4, ACOX1, CBR4, FASN, SCD
Her/Lim	Wnt signaling pathway	10	2.6E-03	COL9A1, SMAD3, WNT1, WNT4, LAMB3, CTNNB1, DVL1, FZD7, NFATC3, IBSP
Her/Lim	ErbB signaling pathway	8	4.9E-04	HRAS, RAF1, SHC1, ERBB2, MAPK3, PRKCA, STAT5A, STAT5B
Her/Lim	mTOR signaling pathway	12	7.7E-05	HRAS, *XBP1*, RAF1, WNT1, WNT4, WNT5A, DVL1, FZD7, GRB10, IGF-1, MAPK3, PRKCA
Her/Lim	PI3K-Akt signaling pathway	16	1.8E-03	HRAS, RAF1, SHC1, SMAD3, MAPK3, STAT5A, STAT5B
Her/Lim	Insulin signaling pathway	9	2.0E-03	HRAS, RAF1, SHC1, FASN, INPPL1, MAPK3, PRKAG3, SLC2A4, SREBF1

Her/Lim	TGF-beta signaling pathway	20	7.7E-05	WNT1, WNT4, WNT5A, CTNNB1, DVL1, MAPK3, STAT5A, STAT5B, NFATC3, PDGFA, PRKCA, STK3, APOA4, APOE, CYP27A1, NCEH1, OSBPL5, CSF1, ERBB2, IGF1R, LRP5
Hf/Lim	Fat digestion and absorption	22	7.5E-18	AGPAT2, ABCG5, ABCG8, NPC1L1, ACAT1, APOA4, APOB, CEL, DGAT1, FABP1, GOT2, PNLIPRP1, PNLIPRP2, PNLIP, PLA2G2C, PLA2G2E, PLA2G2F, PLA2G3, PLA2G5, PLA2G12B, PLPP1, PLPP2
Hf/Lim	Fatty acid metabolism	25	4.4E-20	HACD2,ELOVL2, ELOVL3, ELOVL4, ELOVL5, ACAT1, ACAA2, ACACA, ACADM, ACADSB, ACOX1, ACSBG1, ACSF3,ACSL1, ACSL6, CBR4, CPT1B, CPT1C, CPT2, HADHA, HADHB, HSD17B4, SCD5, SCD, SCP2
Hf/Lim	Cholesterol metabolism	13	9. 0E-08	ABCB11, ABCG5, ABCG8, ANGPTL4, APOA2, APOA4, APOB, APOC2, APOC3, APOE, LDLR, STAR, SOAT2
Hf/Lim	Phospholipase D signaling pathway	23	2.1E-08	AGPAT2, AGPAT4, AGPAT5, HRAS, DGKA, DGKB, DGKI, DGKKINS, PIK3R3, PLA2G4DPLA2G4E, PLA2G4FPLA2G4B, PLCB1, PLCB2, PLCG1, PLCG2, PLD1, PLPP1, PLPP2, PRKCA, SYK
Hf/Lim	Fatty acid degradation	13	3.2E-08	ACAT1, ACAA2, ACADM, ACADSB, ACOX1, ACSBG1, ACSL1, ACSL6, CPT1B, CPT1C, CPT2, HADHA, HADHB
Hf/Lim	Chemokine signaling pathway	14	4.6E-02	HRAS, SRC, WNT1, ADRB1, ADRB2, ADRB3, CYP1A1, CYP1B1, EPHX2, FGF10, MGST2, PIK3R3, PRKC, ARXRA
Hf/Lim	Fatty acid biosynthesis	6	3.1E-04	ACACA, ACSBG1, ACSF3, ACSL1, ACSL6, CBR4
Hf/Lim	TGF-beta signaling pathway	14	1.2E-04	SMAD3, SMAD6, SP1, ACVR1C, BMP2, BMP5, BMP6, BMP7, BMPR1B, GDF6, ID2, TFDP1, TGFB2, TNF
Hf/Lim	Fatty acid elongation	10	4.2E-07	HACD2, ELOVL2, ELOVL3, ELOVL4, ELOVL5, ACAA2, HADHA, HADHB, THEM4, THEM5
Hf/Lim	FoxO signaling pathway	12	7.8E-03	HRAS, SMAD3, CAT, FOXO1, INS, IL10, MAPK14, PCK1, PCK2, PIK3R3, STK11, TGFB2
Hf/Lim	cAMP signaling pathway	15	4.2E-02	NFKBIA, RAC2, ACOX1, ADRB1, ADRB2, FFAR2, GIP, GCG, NPPA,

				PIK3R3, PLCE1, PLD1, POPDC3, PPP1R12A, SSTR5
Hf/Lim	Insulin signaling pathway	11	2.7E-02	HRAS, PPARGC1A, ACACA, FOXO1, INS, PCK1, PCK2, PIK3R3, RHOQ, PRKCI, PKLR
Common	PPAR signaling pathway	6	1.10E-04	HMGCS2, ACOX1, FABP4, PLIN2, RXRA, SCD
Common	Fatty acid metabolism	5	3.50E-04	ELOVL3, ELOVL4, ACOX1, CBR4, SCD
Common	Fat digestion and absorption	4	3.10E-03	AGPAT2, APOA4, PLA2G2F, PLA2G12B
Common	cAMP signaling pathway	6	1.40E-02	ACOX1, ADRB2, GIP, GCG, NPPA, SSTR5
Common	Wnt signaling pathway	5	1.90E-02	SMAD3, WNT1, CTNNB1, NFATC3, PRKCA
Common	PI3K-Akt signaling pathway	7	2.40E-02	HRAS, COL9A1, FGF10, IBSP, LAMB3, PRKCA, RXRA
Common	Lipid and atherosclerosis	5	4.90E-02	HRAS, APOA4, NFATC3, PRKCA, RXRA

Table S5. Gene ontology of differentially expressed genes in Her/Lim, Hf/Lim, and common breeds

Group	Subgroup	GO term	Count	P-value	Gene
Her/Lim	lipid metabolic process	BP	11	2.30E-06	LRP10, ACOX1, FNTB, LACTB, PLCD3, TMEM43, TMEM86A, UMOD, GDPD2, PLCXD3, VLDLR
Her/Lim	cholesterol metabolic process	BP	13	6.20E-13	ABCG1, ERLIN2, LRP5, NFE2L1, APOA4, APOE, CYP27A1, CYP27B1, CYP11B1, FDX1, MBTPS2, SQLE
Her/Lim	skeletal muscle fiber development	BP	5	1.20E-04	SIX1, ACTA1, CACNA1S, MYORG, PLEC
Her/Lim	adipose tissue development	BP	5	1.50E-04	HRAS, LRP5, XBP1, PGRMC2, SELENOM
Her/Lim	fatty acid homeostasis	BP	4	2.20E-04	ABCD1, XBP1, APOE, NR1H4
Her/Lim	skeletal muscle cell differentiation	BP	5	5.20E-04	ATF3, FOXN2, MEF2D, PHOX2B, ZNF689
Her/Lim	response to fatty acid	BP	3	2.60E-03	FOXO3, ILDR1, SCD
Her/Lim	white fat cell differentiation	BP	3	6.60E-03	FABP4, FGF10, ZNF423
Her/Lim	lipid droplet	CC	5	7.70E-03	RAB7A, PNPLA1, PNPLA2, PLIN2, PLIN5
Her/Lim	cytoplasm	CC	58	3.10E-02	BCAR3, FBXO32, GNAZ, GNAS, HDGFL2, KANK2, NAPEPLD, POU4F2, RAF1, SHC1, SIX1, SIX4, TPD52L1, WNT1, WNT4, XBP1, YAP1, ZFP36L2, ACTA1, ACAD10,

					ARRDC3, CTNNB1, CSRP1, DAPK2, ERBB2, EIF2AK2, FABP4, FASN, FGF10, GDF3, GNAZ, IGF1R, IGF2BP1, ITIH4, MC4R, MBTPS2, MAPK3, MEF2D, PPA, NNAT, NFATC3, NCOA2, NR1D1, PRKN, PNPLA1, PNPLA2, PCNT, PLIN5, PID1, PLEC, PRKAG3, PRKCA, SCARB2, STK3, STAT5A, STAT5B, SLC46A1, SORBS3
Her/Lim	Golgi apparatus	CC	18	1.30E-03	NAPEPLD, RAB11B, RAB7A, RAF1, ADRB2, APOE, CTTN, DAPK2, FASN, FUT9, GDF15, INPPL1, MAPK3, MYOC, PRKN, PLD6, SELENOM, SORT1
Her/Lim	Mitochondrion	CC	19	1.90E-02	HMGCS2, ABCG1, GPS2, KANK2, RAB7A, RAF1, CYP27A1, CYP27B1, ECHDC3, FDX1, GPX1, LACTB, NR3C1, PRKN, PLIN5, PLD6, PTGR3, PDK4, SQSTM1
Her/Lim	growth factor activity	MF	8	1.00E-03	BMP5, CSF1, FGF10, GDF15, GDF3, IL6, MSTN, PDGFA
Her/Lim	cholesterol transfer activity	MF	4	1.50E-03	ABCG1, APOA4, APOE, PLTP
Her/Lim	fatty acid binding	MF	4	3.90E-03	ACOX1, FABP4, FABP9, RBP7
Her/Lim	insulin receptor binding	MF	3	1.00E-02	SHC1, GRB10, IGF1R
Her/Lim	phospholipid binding	MF	5	1.30E-02	APOA4, APOE, PLA2G2F, PSAP, PICK1
Her/Lim	fatty acyl-CoA hydrolase activity	MF	11	2.00E-02	ABCD1 ACOT4, ACOT8
Hf/Lim	lipid metabolic process	BP	39	1.90E-29	AGPAT4, AGPAT, BDH2, ATP5F1B, AACS, ASMT, ACP3, ACAD8, ACOX1, AWAT1, AWAT2, ALDH3A1, ANGPTL4, APOC2, B4GALT1, DPEP1, ENPP6, FADS2B, FADS6, FUT1, GDE1, HNF4A, IL1RN, LACTB, LEP, LIPH, MGST2, NPHP3, PNLIPRP1, PNLIP, PLCXD2, PLCXD3, PLCE1, PLCH1, PLCH2, SDS, SCD5, TRPV1, UMOD
Hf/Lim	lipid catabolic process	BP	27	1.30E-21	NAPEPLD, ABHD15, APOA4, APOC2, APOC3, CES1, CLPSL2, ENPP6, LIPH, LIPK, LIPN, OC90, PNLIPRP1, PNLIP,

					PLCXD3, PLA2G2C, PLA2G2E, PLA2G2F, PLA2G5, PLA2G12B, PLCB1, PLCB2, PLCE1, PLCH1, PLCH2, PLD6, PAFAH1B2
Hf/Lim	cholesterol metabolic process	BP	23	2.40E-21	ERLIN1, HNF1A, NSDHL, NFE2L1, APOA2, APOA4, APOA5, APOB, APOE, APOF, CAT, CYP11A1, CYP27B1, CYP27C1, CYP11B1, FDX1, LEP, LDLR, SQLE, STAR, SOAT2, SULT2B1, VLDLR
Hf/Lim	fatty acid metabolic process	BP	22	1.60E-19	ABCD1, ABCD4, ACAT1, ACAA2, ACADM, CPT1B, CPT1C, CPT2, ECHDC2, ECHDC1, HADHA, HADHB, HSD17B10, HSD17B4, LEP, PEX5, PPARD, SLC27, A2SCP2
Hf/Lim	phospholipid metabolic process	BP	19	1.50E-17	GATA6, NKX2-1, ALOX15B, CYP2W1, GDE1, LPCAT2, OC90, PNLIPRP2, PLA2G2C, PLA2G2E, PLA2G2F, PLA2G3, PLA2G5, PLA2G12B, PLB1, PLPPR4, PLPP1, PLPP2, PROCA1
Hf/Lim	fatty acid transport	BP	13	5.00E-14	HNF1A, MFSD2A, CRABP1, FABP1, FABP3, FABP4, FABP6, FABP9, PPARD, RBP1, RBP2, RBP5, RBP7
Hf/Lim	lipid transport	BP	19	1.40E-13	ABCA12, ABCA14, ABCA3, ABCA4, ABCA7, ABCG2, NAXE, VPS51, ANO4, APOC2, APOC3, APOE, APOLD1, ATG9B, ESYT3, GOT2, LBP, SLC25A11, VLDLR
Hf/Lim	fat cell differentiation	BP	16	1.90E-13	ARID5B, C1QTNF3, FOSL2, GLIS1, MKKS, SMAD6, WNT1, WNT3A, ADGRF1, ADGRF5, ALOXE3, EGR2, GDF6, HMGA2, TFAP2B, ZFPM2
Hf/Lim	skeletal muscle cell differentiation	BP	12	4.40E-10	HIVEP3, SMYD1, WNT3A, ATF3, EGR2, GTF3C5, MED20, MYOD1, MYOG, NOTCH1, PHOX2BVAX1
Hf/Lim	adipose tissue development	BP	9	1.10E-07	ARID5B, HRAS, PPARGC1A, RASAL2, ABHD15, LEP, PPARD, PGRMC2, SLC25A25
Hf/Lim	sphingolipid metabolic process	BP	7	1.30E-06	CERKL, GAL3ST1, PSAPL1, PSAP, SMPD2, SFTPB, ZPBP2
Hf/Lim	white fat cell differentiation	BP	3	3.30E-02	FABP4FGF10, FFAR4,

Hf/Lim	lipid droplet	CC	10	6.90E-05	NSDHL, APOB, CES1, CIDEC, LSS, LPCAT2, PNPLA1, PLIN2, PRPF19, RBP1
Hf/Lim	ATP-binding cassette (ABC) transporter complex	CC	2	4.80E-02	ABCG5, ABCG8
Hf/Lim	lysosome	CC	17	6.50E-05	CD1B, SRC, TCIRG1, CD1B5, WWOX, ACP3, ARRDC3, LAMTOR1, LDLR, MLC1, PRDX6, PSAPL1, PSAP, SCARB2, SFTPB, TLR9, VPS4A
Hf/Lim	fatty acid binding	MF	14	1.00E-13	ACOX1, ACOXL, CRABP1, FABP1, FABP3, FABP4, FABP6, FABP9, FFAR4, HNF4A, RBP1, RBP2, RBP5RBP7,
Hf/Lim	lipid binding	MF	18	3.20E-09	BPIFA1, BPIFA3, BPIFA2B, BPIFA2C, BPIFB1, BPIFB2, BPIFB3, BPIFB4, BPIFB6, NFE2L1, STARD13, ACOT12, APOC2, APOE, APOLD1, ESYT3, FABP3, SCP2,
Hf/Lim	cholesterol binding	MF	13	3.30E-09	ERLIN1, GRAMD1A, GRAMD1B, NPC1L1, APOA2, APOA4, APOA5, OSBPL6, OSBPL7, SCARB2, STAR, SOAT2, SULT2B1
Hf/Lim	phospholipid binding	MF	14	1.90E-07	APOA4, APOA5, APOB, APOC3, APOE, BIN1, DGKA, OC90, PLA2G2C, PLA2G2E, PLA2G2F, PLA2G5, PSAP, PRKCI,
Hf/Lim	lipid transporter activity	MF	6	7.60E-05	ABCA12, ABCA14, ABCA3, ABCA4, ABCA7, NPC1L1
Hf/Lim	calcium ion binding	MF	33	4.40E-04	ALOX15B, DGKA, DGKB, ESYT3, LDLR, LPCAT2, NAPEPL D, NOTCH1, OC90, PITPNM2, PLA2G12B, PLA2G2C, PLA2G2E, PLA2G2F, PLA2G4D, PLA2G4E, PLA2G4F, PLA2G5, PLCB1, PLCB2, PLCG1, PLCH1, PLCH2, PNLIPRP2, PPP3CB, SULF2, VLDLR, VSNL1
Hf/Lim	growth factor activity	MF	12	1.30E-03	BMP10, BMP2, BMP3, BMP5, BMP6, BMP7, FGF10, GDF15, GDF3, GDF6, MSTN, TGFB2,
Common	lipid catabolic process	BP	8	2.50E-08	NAPEPLD, APOA4, CLPSL2, LIPK, PLCXD3, PLA2G2F, PLA2G12B, PLD6

Common	cholesterol metabolic process	BP	7	5.10E-08	NFE2L1, APOA4, ZAPOE, CYP27B1, CYP11B1, FDX1, SQLE,
Common	long-chain fatty acid transport	BP	4	1.90E-06	APOE, FABP4, FABP9, PLIN2
Common	cholesterol homeostasis	BP	5	8.80E-05	APOA4, APOE, FABP4, NR1H4, PLA2G12B
Common	fatty acid homeostasis	BP	3	7.80E-04	APOA4, APOE, FABP4, NR1H4, PLA2G12B
Common	adipose tissue development	BP	2	4.80E-02	HRAS, PGRMC2
Common	endoplasmic reticulum membrane	CC	8	1.30E-02	ABCD1, ELOVL3, ELOVL4, PEDS1, SHH, SQLE, SCD
Common	extracellular space	CC	18	3.20E-05	ADAMTS4, WNT1, APOE, COL3A1, COL9A1, FGF10, GIP, GCG, GDF15, GDF3, IBSP, IFNT3, IFN-TAU, IL10, IL17A, IL36G, NPPA, SHH
Common	euchromatin	CC	3	5.20E-03	POU4F2, SP1, NR1H4
Common	fatty acid binding	MF	3	5.00E-03	ACOX1, FABP4, FABP9
Common	long-chain fatty acid binding	MF	2	2.90E-02	FABP4, FABP9
Common	fatty acid elongase activity	MF	2	2.90E-02	ELOVL3, ELOVL4
Common	phospholipid binding	MF	3	3.60E-02	APOA4, APOE, PLA2G2F
